# Antimicrobial stewardship auditing of patients reviewed by infectious diseases physicians in a tertiary university hospital

**DOI:** 10.1186/2047-2994-2-29

**Published:** 2013-11-01

**Authors:** Chay Leng Yeo, Jia En Wu, Gladys Wei-Teng Chung, Douglas Su-Gin Chan, Hui Hiong Chen, Li Yang Hsu

**Affiliations:** 1 Department of Pharmacy, National University Health System, 5 Lower Kent Ridge Road, Singapore 119074, Singapore; 2 Department of Laboratory Medicine, National University Health System, 5 Lower Kent Ridge Road, Singapore 119074, Singapore; 3 Department of Medicine, National University Health System, NUHS Tower Block Level 10, 1E Kent Ridge Road, Singapore 119228, Singapore; 4 Saw Swee Hock School of Public Health, National University of Singapore, MD3, 16 Medical Drive, Singapore 117597, Singapore

**Keywords:** Antimicrobial stewardship, Infectious diseases physicians, Conflicts, Antimicrobial resistance, Compliance

## Abstract

**Background:**

The optimal way for antimicrobial stewardship programs (ASPs) to interact with existing infectious disease physician (IDP) services within the same institution is unknown. In our institution, IDPs and our prospective audit and feedback ASP operate independently, with occasionally differing recommendations offered for the same inpatient. We performed a retrospective audit on inpatients that had been reviewed by both IDPs and ASP within a 7-day period, focusing on cases where different therapy-modifying recommendations had been offered. We analyzed the outcomes in inpatients where the ASP recommendations were accepted and compared these with the inpatients where the IDP recommendations were accepted instead. Outcomes assessed were 30-day mortality post-ASP review, unplanned re-admission within 30 days post-discharge from hospital, and clinical deterioration at 7 days post-ASP review.

**Findings:**

There were 143 (18.9%) patients where differing recommendations had been offered, with primary physicians accepting 69.9% of ASP recommendations. No significant differences in terms of demographics, clinical characteristics, 30-day mortality, and re-admission rates were observed, although clinical deterioration rates were lower in patients where the ASP recommendation was accepted (8.0% vs. 27.9%; *p* = 0.002). On multivariate analysis, hematology-oncology inpatients were associated with unplanned readmission. Increasing age and hematology-oncology inpatients were associated with clinical deterioration 7 days post-recommendation, whereas acceptance of ASP recommendations was protective. No characteristic was independently associated with 30-day mortality.

**Conclusion:**

In conclusion, independent reviews by both IDPs and ASPs can be compatible within large tertiary hospitals, providing primary physicians even in situations of conflicting recommendations viable alternative antimicrobial prescribing advice.

## Findings

### Introduction

Infectious diseases (ID)-trained physicians are considered integral to antimicrobial stewardship programs (ASPs), conferring program legitimacy with regards to other hospital physicians and ensuring that ASP activities do not put patients at greater risk of adverse outcomes [1]. However, there can be considerable variability in the antibiotic prescribing practices of ID physicians [2,3], particularly if they had received training at different institutions.

In institutions with both an ID service and an ASP, it is inevitable that broad-spectrum antibiotics prescribed to patients by their primary physicians based on advice by ID physicians will come under the ambit of the ASP. It is also inevitable that there will be differences between ID physicians’ and the ASP’s clinical interpretations with respect to antibiotic prescribing in a subset of these cases. How an ASP should function in such situations has not been described in the medical literature, although three main courses of action are apparent:

• Disregard all patients where an ID physician’s clinical input has been sought.

• Review such patients, but contact the ID physician should the ASP’s view not coincide with the ID physician’s recommendations, and come to an agreed recommendation.

• Review such patients and submit an ASP recommendation independently.

Each approach has its advantages and disadvantages in terms of oversight, clinical authority (of both ID physicians and the ASP), impact of the ASP on antibiotic prescribing rates, and collegiality. In our institution, where a prospective audit and feedback ASP has been established since 2009 [4], we have consistently taken the last approach. We present herein our experience where ASP and ID physicians provide independent assessments and recommendations, analyzing the outcomes of a large subset of cases where the recommendations have differed.

## Setting and methods

We performed a retrospective analysis on all patients reviewed concurrently by ID physicians and the ASP between 1^st^ August 2011 and 30^th^ November 2012 at our institute – a 994-bed tertiary university hospital. ID physicians provide a standard referral service whereas the prospective audit and feedback system of our ASP is also standard and has been described elsewhere [4,5]. Clinical and demographic data were extracted from our ASP database as well as the hospital electronic records. The Charlson comorbidity index was used as an aggregate measure for patients’ comorbidity prognostication [6].

The unit of measurement was a patient that had been reviewed within one week by both an ID physician and the ASP. Patients where differing therapy-modifying recommendations were made by the ID physician and the ASP were selected for more in-depth analysis of outcomes. Outcome measures include 30-day mortality post-ASP review, unplanned re-admission within 30 days post-discharge from hospital, and clinical deterioration at 7 days post-ASP review – a composite measure comprising persistence of fever (if febrile), failure of microbiological clearance, and lack of general physiological improvement defined by the primary physicians’ records.

Intercooled Stata 12.1 (StataCorp, College Station, USA) was used for all statistical calculations, with the level of significance set at 5%. For determination of covariates associated with the various outcomes, we performed logistic regression, with a multivariate model based on covariates identified in the univariate analysis (*p*-value < 0.200) using the likelihood ratio test, with probability of removal set at 0.05.

The NUH Medical Board approved this work. Informed consent was not obtained from individual patients as both the operations of ID physicians and the ASP constituted routine clinical practice, and only anonymized data were analyzed.

The National University Health System funded this work internally and had no role in study design, analysis or write-up of the results.

## Results

There were 756 patients within the audit period that had concurrently been reviewed by both ID physicians and the ASP, of which 143 (18.9%) had differing therapy-modifying recommendations. The clinical, demographic and outcome data for these patients are shown in Table 1, classified by whether ASP recommendations (100 cases; 69.9%) or ID physicians’ recommendations were accepted. In the majority of these cases, ID physicians generally recommended continuation of broad-spectrum antibiotics rather than de-escalation, longer duration of antibiotics, or combination antibiotics (particularly for *Pseudomonas aeruginosa* infections).

**Table 1:** Demographic, clinical and outcome data of 143 patients with differing therapy-modifying recommendations by ASP and ID physicians

	**ASP recommendation accepted**	**ID physician recommendation accepted**	***p*-value**
	**(*n *** **= 100)**	**(*n *** **= 43)**	
Median age, years (interquartile range)	64 (53–72)	58 (50 – 72)	0.393
Intensive care unit admission within 30 days prior to review (%)	23 (23.0)	10 (23.3)	0.973
Median Charlson Comorbidity Index (interquartile range)	5 (3 – 8)	5 (2 – 8)	0.255
Clinical discipline (%)			0.754
• Hematology-oncology	36 (36.0)	12 (27.9)	
• Surgery	29 (29.0)	12 (27.9)	
• Orthopedics	24 (24.0)	13 (30.2)	
• Medicine	10 (10.0)	6 (14.0)	
• Others	1 (1.0)	0 (0)	
Type of infection (%):			0.247
• Intra-abdominal infection	23 (23.0)	9 (20.9)	
• Bloodstream	12 (12.0)	6 (14.0)	
• Bone and joint infection	14 (14.0)	12 (27.9)	
• Skin and soft tissue infection	15 (15.0)	6 (14.0)	
• Respiratory tract infection	6 (6.0)	2 (4.7)	
• Urinary tract infection	10 (10.0)	6 (14.0)	
• Others^a^	20 (20.0)	2 (4.7)	
Type of ASP recommendations:^b^			0.172
• Discontinue antibiotics	26	5	
• De-escalate antibiotics	30	16	
• Escalate antibiotics	8	5	
• Intravenous to oral antibiotic switch	6	2	
• Dose optimization	16	5	
• Duration of antibiotics	0	2	
• Discontinue duplicate antibiotic coverage	9	7	
• Other recommendations^c^	10	3	
Outcomes (%):			
• 30-day mortality	7 (7.0)	3 (7.0)	1.000
• 30-day re-admission^d^	21 (22.6)	6 (15.0)	0.358
• Clinical deterioration	8 (8.0)^e^	12 (27.9)^f^	0.003
○ Persistent fever	2 (25.0)	4 (33.3)	
○ No microbiological clearance	2 (25.0)	3 (25.0)	
○ Lack of physiological improvement	6 (75.0)	10 (83.3)	

^a^"Others" include undifferentiated fevers, viral infections, and no infection.

^b^There were 150 ASP recommendations for 143 patients.

^c^"Other recommendations" include switching to less expensive carbapenems (9 cases), streamlining antibiotics (2 cases) and addition of antifungal agents (2 cases).

^d^Patients who had died are excluded.

^e^One patient had persistent fever and bacteremia; one patient had persistent fever with hypotensive episodes; one patient had persistent candidemia with respiratory distress.

^f^Two patients had persistent fever and persistent positive wound cultures; one patient had persistent fever and bacteremia with cardiac failure; one patient had persistent fever with hypotension.

The majority of patients were from the surgical disciplines (54.5%) or the hematology-oncology department (33.6%). There was no significant difference in terms of demographic or clinical characteristics for patients where the ASP recommendations had been accepted or rejected in favor of ID physicians’ recommendations, although physicians of patients with bone and joint infections had higher rates of acceptance of ID physicians’ recommendations, with the converse being true for physicians of patients with undifferentiated fevers or where no infection was present.

There was no difference in 30-day mortality or 30-day re-admission rates between the two groups, although clinical deterioration rates were lower in patients where the ASP recommendations had been accepted. Six of 10 deaths occurred in patients with advanced cancers who were on palliative care.

The results of univariate analysis of demographic and clinical characteristics with outcomes are shown in Table 2. Multivariate analysis was not performed for 30-day mortality as none of the demographic or clinical characteristics had a *p*-value < 0.200 on univariate analysis. The only characteristic associated with unplanned readmission 30 days post-recommendation was being from the hematology-oncology department (OR = 4.133; 95% CI: 1.301 – 13.122; *p* = 0.016). Increasing age (OR = 1.043; 95% CI: 1.003 – 1.085; *p* = 0.037) and being from the hematology-oncology department (OR = 6.116; 95% CI: 1.352 – 27.665; *p* = 0.019) was associated with clinical deterioration 7 days post-recommendation, whereas acceptance of ASP recommendations (OR = 0.151; 95% CI: 0.050 – 0.457; *p* = 0.001) was protective.

**Table 2:** Univariate analysis of the impact of cohort characteristics with outcomes

**Characteristic**	**30-day mortality**			**30-day re-admission**			**Clinical deterioration at 7 days**		
	**Odds ratio**	**95% confidence interval**	***p-*** **value**	**Odds ratio**	**95% confidence interval**	***p-*** **value**	**Odds ratio**	**95% confidence interval**	***p-*** **value**
Higher age	1.015	0.968 – 1.064	0.545	1.021	0.989 – 1.055	0.198	1.027	0.990 – 1.064	0.155
Prior ICU^a^ admission	0.351	0.043 – 2.875	0.329	0.975	0.355 – 2.725	0.975	0.547	0.150 – 1.996	0.361
Higher Charlson co-morbidity index	1.064	0.871 – 1.301	0.542	1.033	0.897 – 1.190	0.653	1.028	0.884 – 1.194	0.724
Clinical discipline (relative to surgery)									
• Hematology-oncology	N.A.*	N.A.*	N.A.*	3.855	1.241 – 11.980	0.020	3.462	0.875 – 13.693	0.077
• Orthopedics	0.452	0.085 – 2.390	0.265	1.720	0.453 – 6.530	0.426	3.333	0.770 – 14.436	0.107
• Medicine	1.273	0.225 – 7.197	0.792	1.911	0.319 – 11.450	0.478	2.727	0.405 – 18.358	0.302
• Others	N.A.*	N.A.*	N.A.*	N.A.*	N.A.*	N.A.*	N.A.*	N.A.*	N.A.*
Type of infection (relative to intra-abdominal infections)									
• Bloodstream	0.882	0.074 – 10.464	0.921	0.438	0.080 – 2.400	0.342	1.080	0.226 – 5.162	0.923
• Bone and joint infection	1.957	0.302 – 12.692	0.482	0.493	0.112 – 2.164	0.349	1.286	0.329 – 5.032	0.718
• Skin and soft tissue infection	1.579	0.205 – 12.173	0.661	0.616	0.138 – 2.749	0.526	N.A.*	N.A.*	N.A.*
• Respiratory tract infection	N.A.*	N.A.*	N.A.*	1.095	0.179 – 6.694	0.922	0.771	0.077 – 7.712	0.825
• Urinary tract infection	1.000	0.084 – 11.931	1.000	0.548	0.098 – 3.057	0.492	1.246	0.257 – 6.031	0.784
• Others	0.750	0.064 – 8.834	0.819	1.095	0.293 – 4.097	0.526	0.900	0.191 – 4.243	0.894
ASP recommendation accepted	1.004	0.247 – 4.079	0.996	1.412	0.517 – 3.859	0.501	0.225	0.084 – 0.600	0.003

^a^ICU = intensive care unit.

^*^N.A. = predicted failure of regression perfectly (none or all patients in this category).

## Discussion

The most effective way to implement antimicrobial stewardship is not known at this point in time [7], but is probably strongly influenced by local practices, history and culture. Both ID physicians (not involved in antimicrobial stewardship) and ASPs should ideally support the concept of antimicrobial stewardship and appropriate prescription of antimicrobial agents [1,7]. However, there may be differences in the perceived importance of various aspects of clinical care, leading at times to radically different antimicrobial prescribing recommendations. As an example, institutional antimicrobial resistance rates and the differential costs of antibiotics may matter far less to an ID physician than an ASP, resulting in a more conservative approach to de-escalating or stopping antibiotics in patients that have more complications or a slower clinical response, regardless of culture results.

In an institution with both ID physicians and an ASP, there may also be professional friction if one is seen to encroach on the clinical domain of the other. Patients’ primary physicians may also be confused if conflicting recommendations were received from the ID physicians and the ASP. In the majority of local institutions, the ASPs do not audit patients who are under the active review of an ID physician (data not shown). Nonetheless, there may be benefits for patients – particularly in large clinical institutions with high patient workload – if ASPs and ID physicians provide independent reviews and cross-checks, providing an answer to the perennial question of "quis custodiet ipsos custodes" (who shall watch the watchmen) in the context of antimicrobial prescribing and stewardship. Our results suggest that such an approach can be complementary. Concordant recommendations were made for the vast majority of patients reviewed by both ASP and ID physicians. Where recommendations have differed, patients did not have worse outcomes when the ASP’s recommendations were preferred. Although patients where the ASP recommendations were accepted appeared to have better clinical response at Day 7 post-recommendation, it is plausible that this is confounded by primary physicians preferring ID physicians’ recommendations over ASP recommendations in the setting where patients had been more ill or were clinically deteriorating. Unfortunately there were insufficient data and patients to clarify this. Anecdotally, primary physician confusion was minimal, with most teams recognizing the different roles and review processes of the ID physicians and the ASP.

A compromise approach would be to initiate discussions between ID physicians and the ASP each time there is a disagreement about antimicrobial prescribing recommendations. This will reduce although probably not eliminate conflicting recommendations to the patients’ primary physicians, and may be ideal in most institutional settings where the clinical workload is not overwhelming.

Our other results are unsurprising in finding that higher age and having a hematological or oncological (the patients mostly had hematological malignancies) condition were associated with subsequent clinical deterioration, and most unplanned admissions for patients from this department were for the development of febrile neutropenia post-chemotherapy. Because data for calculating acute physiological scores were not collected as part of the study, and the number of deaths was relatively small, there were no significant co-variates associated with 30-day mortality found.

Our audit is primarily limited by the relatively small patient numbers and the methodology. The definition of clinical deterioration was also very broad and data collection for this variable was performed as a routine function of the ASP (and therefore not blinded with respect to whether ASP recommendations were accepted). Nonetheless, the results appear robust and it is difficult if not impossible to run a prospective study within a single institution – perhaps cluster randomization will be required to determine if an approach of independent reviews and recommendations by both ID physicians and ASPs will positively benefit clinical care. We have also not rigorously audited the results in terms of the cost-savings of ASP recommendations vis-à-vis ID physicians’ recommendations. One final major limitation of this audit is that the findings may not be applicable outside of our institution or country, as cultural differences may significantly affect the success or failure of such practices.

In conclusion, we have demonstrated that independent reviews by both ID physicians and ASPs can be compatible within a large tertiary university hospital, providing primary physicians even in situations of conflicting recommendations viable alternative antimicrobial prescribing recommendations. This approach will also not result in social or professional conflict between ID physicians and ASPs provided that communication is good. Initiating early discussions between ID physicians and ASPs in situations where antimicrobial prescribing advice differs may significantly reduce the number of conflicting recommendations.

## Competing interests

LYH has received research funding and speaker’s honoraria from Pfizer, AstraZeneca, Janssen & Cilag, and Merck, Sharpe & Dohme. Other authors have no conflicts of interest to declare.

## Authors’ contributions

CLY and LYH conceived the study and analyzed the data. CLY, JEW, GWC, HHC and DSC acquired the data. CLY drafted the manuscript, while critical revision was provided by HLY. All authors read and approved the final version of the manuscript.
